# Elevated HMGB1 promotes the malignant progression and contributes to cisplatin resistance of non-small cell lung cancer

**DOI:** 10.1186/s41065-023-00294-9

**Published:** 2023-07-31

**Authors:** Ying Ma, Qin Feng, Bateer Han, Rong Yu, Zhiyong Jin

**Affiliations:** 1 Department of Thoracic Surgery, Affiliated People’s Hospital of Inner Mongolia Medical University, No.1 Tong Dao Bei Road, Hohhot, 010059 China; 2 Inner Mongolia Cancer Hospital and Affiliated People’s Hospital of Inner Mongolia Medical University, Hohhot, 010020 China

**Keywords:** High mobility group B1 (HMGB1), Non-small cell lung cancer (NSCLC), Cisplatin, Multidrug resistance (MDR)

## Abstract

**Background:**

HMGB1 (high mobility group box B-1) exhibits crucial role in tumor genesis and development, including lung cancer. Whereas, more HMGB1-related details in non-small cell lung cancer (NSCLC) are still largely unclear.

**Methods:**

The HMGB1 and inflammatory factors in malignant (MPE) and non-malignant pleural effusion (BPE) were determined by ELISA. Additionally, qRT-PCR, western blot, or immunohistochemistry were used to determine HMGB1, drug-resistant and apoptotic proteins’ expressions in NSCLC A549, A549-DDP cell lines, and xenograft model. Cell viability, migration/ invasion, and apoptosis were analyzed using MTT, Transwell, and flow cytometry assays, respectively.

**Results:**

Inflammatory factors and HMGB1 expressions in MPE were significantly higher than BPE of NSCLC. Compared with preoperative and adjacent tissues, significantly higher HMGB1, drug-resistant protein, and anti-apoptotic protein expressions were observed in recurrent tissues. Overexpressed HMGB1 induced NSCLC cells to exhibit stronger aggressive, proliferative, and drug-resistant features. The related abilities were reversed when HMGB1 was interfered. Overexpressed HMGB1 showed a similar co-localization with drug resistant protein P-gp in cytoplasm in xenograft model, while low HMGB1 expression localized in cell nucleus.

**Conclusions:**

HMGB1 overexpression significantly promoted the malignant progression and cisplatin resistance of NSCLC in vitro and in vivo.

**Supplementary Information:**

The online version contains supplementary material available at 10.1186/s41065-023-00294-9.

## Introduction

Lung cancer has been the most frequent cancer with the highest mortality, causing approximately an estimated 1.8 million deaths according to the latest global cancer statistics [1]. Among the lung cancer cases, about 80–85% patients are diagnosed with non-small cell lung cancers (NSCLCs) [2, 3]. Despite rapid development in chemotherapy and immunotherapy of NSCLC, the clinical outcomes of NSCLC patients are still unsatisfactory, especially facing the fact of over 40% patients diagnosed at advanced stages (stage IIIB or IV) [4]. Meanwhile, the chemo-resistance and distant metastasis of patients remain the predominant obstacles for NSCLC treatment and prognosis improvement. Currently, the chemoradiation is still contained in the optimal treatment strategy for NSCLC patients, which is usually combined with other therapies based on the actual conditions [5]. Cisplatin is a frequently used chemotherapy drug in the treatment of NSCLC. It has been indicated that the anticancer effects of cisplatin can be achieved through damaging nuclear DNA [6], but the DNA repair mechanisms in patients promote the acquisition of cisplatin resistance in tumor cells [7]. Accordingly, most NSCLC patients could not successfully finish the chemotherapy due to drug resistance [8–10]. Furthermore, the detailed mechanisms underlying cisplatin resistance in NSCLC remain to be clarified as far as we know, which has greatly limited the longterm therapeutic efficacy. Hence, it is imperative and necessary to demonstrate the potential mechanisms behind drug resistance in order to discover novel efficacious therapies for NSCLC patients and thereby improve the prognosis.

High mobility group box (HMG) is a typical class of non-histone proteins with molecular weight of about 25 KD in the nucleus [11]. HMG consists of three families: HMGA, HMGB, and HMGN, the most evolutionarily conserved of which is HMGB1 (high mobility group box B-1) [12, 13]. HMGB1 widely exists in various human tissues, and the function of HMGB1 often varies depending on its distribution. Moreover, HMGB1 is able to bind DNA in the nucleus and help specific DNA-binding proteins to correctly operate to the binding sites on chromosomes [14, 15]. The earliest HMGB1 related studies have mainly concentrated on the correlation between HMGB1 protein and inflammatory response. For instance, TLR2 and TLR4, as the main receptors of HMGB1 protein, play an important role in extracellular inflammatory response and intensify the inflammatory response through activating NF-κB signaling pathway [16, 17]. HMGB1 could also locate injury and promote aggregation of inflammatory factors to exacerbate the injury. Recent studies have documented that HMGB1 can involve in a variety of tumors and autoimmune diseases [18]. It has been suggested that there will be higher serum HMGB1 contents at later clinical stages [19]. Meanwhile, some recent studies have revealed the role of HMGB1 in various tumors. For instance, HMGB1 could affect the cell invasion and PD-L1 expression by regulating RAGE-PI3K/AKT signaling pathway in breast cancer cells [20]. Besides, Chen et al. have documented the castration-resistant promoting role of HMGB1 in prostate cancer via regulating androgen receptor activation [21]. However, the study of HMGB1 in NSCLC is still in its early stage, and there is a lack of systematic reports on the detailed roles of HMGB1 in cisplatin resistance in NSCLC, as well as the correlation between HMGB1 and various clinicopathological features.

Accordingly, our present work aimed to elucidate the potential role of HMGB1 in the progression and cisplatin resistance of NSCLC, integrating clinical samples, cell lines and in vivo experiments. Our findings associating with HMGB1 and cisplatin resistance are promising to be further explored and applied in clinical cases, as a novel therapeutic target in NSCLC treatment.

## Materials and methods

### Patients and specimen preparation

From November 2014 to April 2016, totally 23 pairs of NSCLC vs. adjacent samples, 46 cases of malignant pleural effusion (MPE) samples, and 31 cases of non-malignant pleural effusion (BPE) samples were randomly collected from the Affiliated Hospital of Inner Mongolia Medical University and Nanfang Hospital of Southern Medical University. All participants have provided the informed consents. Our study was approved by ethic committee of Affiliated Hospital of Inner Mongolia Medical University and Nanfang Hospital of Southern Medical University, in accordance with The Helsinki Declaration. Prior to pleural effusion or lung tissue sample collection, neither chemotherapy nor radiation therapy had been administered to NSCLC patients. All tissue specimens and pleural effusion specimens were promptly frozen and stored at -80℃.

### Cell culture and transfection

The NSCLC cell line A549 and cisplatin-resistant NSCLC cell line A549-DDP were provided by Southern Medical University (Guangzhou, China). A549 and A549-DDP cell lines were cultured in Dulbecco’s modified eagle medium (DMEM) (GIBCO, Brazil) supplementing with 10% fetal bovine serum (FBS; GIBCO, Brazil), in a 37 °C with 5% CO_2_ incubator. Lipofectamine 2000 (Invitrogen, USA) was used to transfect A549 cells and A549-DDP cells. Cells were used for subsequent research after 18–48 h of transfection. The primer sequences employed were contained in Table S1. pcDNA 3.1-HMGB1 and si-HMGB1 were obtained from RiboBio (Guangzhou, China). Negative controls included the empty vector (pcDNA 3.1), si-NC, mimics NC, and inhibitor NC (NC).

### ELISA

The protein levels of IL-6 (Abcam, UK), IL-8 (Abcam, UK), IL-1β (Cyagen, China) and HMGB1 (BIOVISION, USA) in pleural effusion samples were analyzed using ELISA kits. The assay was completed strictly following the manufacturer’s instructions. The OD value at 450 nm was monitored with a Perlong DNM-9602 Microplate reader (Beijing, China).

### Cell viability analysis

Cells were planted in 96-well plates at a density of 5 × 10^3^ cells per well with or without transfection for 24 h and then administrated with cisplatin (0, 0.5, 5, 15, 30, 60 µmol/L), docetaxel (0, 0.5, 5, 15, 30, 60 µmol/L), gemcitabine (0, 0.5, 5, 15, 30, 60 µmol/L), pemetrexed (0, 0.5, 1, 3, 5, 7 µmol/L) and paclitaxel (0, 0.5, 3, 6, 9, 12 µmol/L) for 24 h, 48 h, 72 h. An 3-(4, 5-dimethylthiazol-2-yl)-2, 5-diphenyl tetrazolium bromide (MTT) Assay Kit (Sigma, USA) was used to determine the cytotoxicity and cell viability. Based on the absorbance at 490 nm measured in a microplate reader (Tecan Group Ltd., Switzerland), the half maximum inhibitory concentration (IC50) was calculated. The formula of Drug resistance index = mean IC50 of drug-resistant cell lines/ mean IC50 of sensitive cell lines.

### Colony formation assay

Six-well plates were planted with resuspended A549 cells and A549-DDP cells (1 × 10^3^ cells/mL). After the culture at 37 °C for 14 days, the cells were stained with 0.1% crystal violet and 20% methanol, and the number of cell colonies was then calculated.

### Migration and invasion assay

Migration and invasion tests were conducted using Transwell assays. For the migration tests, 200 µL (1 × 10^6^ cells/mL) of cell suspension media was placed in the top chamber, and complete medium was added to the lower chamber. Cells were cultured for 24 h at 37 °C with 5% CO_2_ before being fixed with 4% paraformaldehyde and stained with 0.1% crystal violet solution. Regarding the invasive ability of the cells, Transwell chambers precoated with Matrigel (BD Bioscience, San Jose, CA, USA) was used. Briefly, 200 µL (1 × 10^6^ cells/mL) of cell suspension were added into the upper chambers, and the DMEM with 10% FBS as a chemoattractant was plated into the lower chambers. After culture for 24 h at 37 °C with 5% CO_2_, the invasive cells in the lower chambers were fixed with 4% paraformaldehyde for 30 min and stained with 0.1% crystal violet for 5 min at room temperature. The migration and invasive cells were both counted under an inverted light microscope.

### Apoptosis analysis

A flow cytometer (FACSCalibur, BD, USA) was utilized to measure cell apoptosis according to the manufacturer’s protocols. Following treatment, the cells were labeled with FITC and PI, and the apoptosis rate of cells in various groups was examined by flow cytometry. FlowJo V10 software (Tree Star, USA) was used to analyze the data.

### Mouse xenograft model

All 50 BALB/c mice (6–7 weeks of age) were purchased from the Beijing Wei Shang Lide Biological Technology (Beijing, China), and then were kept under pathogen-free environment. The 50 BALB/c mice were randomly divided into 7 groups, including 6 experimental groups (8 per group) and a model identification group (n = 2). Regarding the 6 experimental groups, the A549, A549-PCDNA3.1-HMGB1, A549-PCDNA3.1 NC, A549-DDP, A549-DDP siRNA NC, and A549-DDP Si-HMGB1 cells were subcutaneously inoculated into the left flanks of the mice (n = 8 per group). The tumor volumes were measured and estimated by the formula volume = length(width/2)2 every three days. The model identification group was used for pathological examination to confirm the tumor model successful construction. Mice in each group were randomly divided into two groups, intraperitoneal injection of cisplatin and paclitaxel, respectively (cisplatin: 1.5 mg/kg, paclitaxel: 20 mg/kg, 4 mice per group, once a day). 21 days later, all mice were killed and the tumors were collected immediately. All animal experiments in this study were conducted according to the institutional guidelines of the Animal Care and Use Committee at Nanfang Hospital of Southern Medical University.

### Immunohistochemistry (IHC)

Following heat-activated antigen extraction, sections were stained immunohistochemically according to the preceding description [22]. The following primary antibodies were used: HMGB1 (ab79823, Abcam, UK, 1:400), Bax (ab32503, Abcam, UK, 1:250), Bcl-2 (ab182858, Abcam, UK, 1:400), lung resistance protein (LRP, ab92544, Abcam, UK, 1:100), multidrug resistance-related protein (MRP, ab260038, Abcam, UK, 1:500), P-glycoprotein (P-gp, ab170904, Abcam, UK, 1:500), and the secondary antibody, HRP Anti-Rabbit IgG antibody (Abcam, UK, 1:600), was utilized. Images were taken with an Olympus BH2 microscope (Olympus Corporation, Japan).

### Immunofluorescence

Briefly, the sections were treated with Triton X-100 and blocked with goat serum. Then, the primary antibody (HMGB1, ab79823, Abcam, UK, 1:400; P-gp, ab170904, Abcam, UK, 1:500) was used to incubate the cells, followed by a secondary antibody (HRP Anti-Rabbit IgG antibody, Abcam, UK, 1:600) incubation. After DAPI staining, the localization was viewed under a confocal laser scanning microscope (Olympus, Japan).

### TUNEL assay

Following the manufacturer’s protocols, the TUNEL apoptosis test kit-FITC (Boster, Wuhan, China) was used to determine the cell apoptosis levels in tumor tissues. 4% paraformaldehyde was used to fix frozen portions for 1 h, followed by 10 min of proteinase K digestion.

### Hematoxylin-eosin (HE) staining

Frozen portions were fixed with pre-cooled acetone after 10 min at room temperature (RT). It was distinguished with 1% hydrochloric acid alcohol after 5 min of hematoxylin staining. Sections were then sealed after being transparentized by xylene and dehydrated with alcohol. Pictures were taken with an Olympus BH2 microscope (Olympus, Japan).

### Quantitative real-time polymerase chain reaction (qRT-PCR)

Using the Trizol reagent (TakaRa, Japan), total RNA was extracted from tissue or cells. The M-MLV (TakaRa, Japan) was used to reverse-transcribe a total of 1 g of RNA in a final volume of 30 µL. qRT-PCR with the SYBR® Green mastermix (TakaRa, Japan) on iQ5 Real-Time PCR System (BioRad, USA). The PCR primers were listed as follows: HMGB1 forward: 5’-ATGCGCAAAGGAGATCCTA-3’, reverse: 5’-ATTCATCATCATCTTCT-3’, and GAPDH forward: 5’-ACAGTCAGCCGCATCTTCTT-3’, reverse: 5’-GACAAGCTTCCCGTTCTCAG-3’.

### Western blot

Polyvinylidene difluoride (PVDF) membranes were used to transfer the whole protein separated with SDS-PAGE. The primary antibody was added and incubated at 4 °C for overnight and the secondary antibody was incubated at 37 °C for an hour after non-fat milk powder blocked the PVDF membrane for an hour at RT. All antibodies we used were shown as below: HMGB1 (ab79823, Abcam, UK, 1:5000), Caspase-3 (ab184787, Abcam, UK, 1:2000), Bax (ab32503, Abcam, UK, 1:1000), Bcl-2 (ab182858, Abcam, UK, 1:2000), LRP (ab92544, Abcam, UK, 1:5000), MRP (ab260038, Abcam, UK, 1:1000), P-gp (ab170904, Abcam, UK, 1:1000), and the secondary antibodies (HRP Anti-Rabbit IgG antibody, Abcam, UK, 1:8000). Chemiluminescence substrate (Boster, China) was used to determine the gray value.

### Statistical analysis

Data were presented as mean and standard. The statistical program GraphPad Prism (v9.5.0, GraphPad Software Inc, La Jolla, USA) was utilized for all analyses. The two-tailed Student t test was utilized for statistical comparisons between two groups. One-way ANOVA was used for difference significant evaluation among three groups, followed by a Tukey’s post hoc test. P<0.05 was considered statistically significant.

## Results

### HMGB1 significantly elevated in malignant pleural effusion

**Fig. 1:**
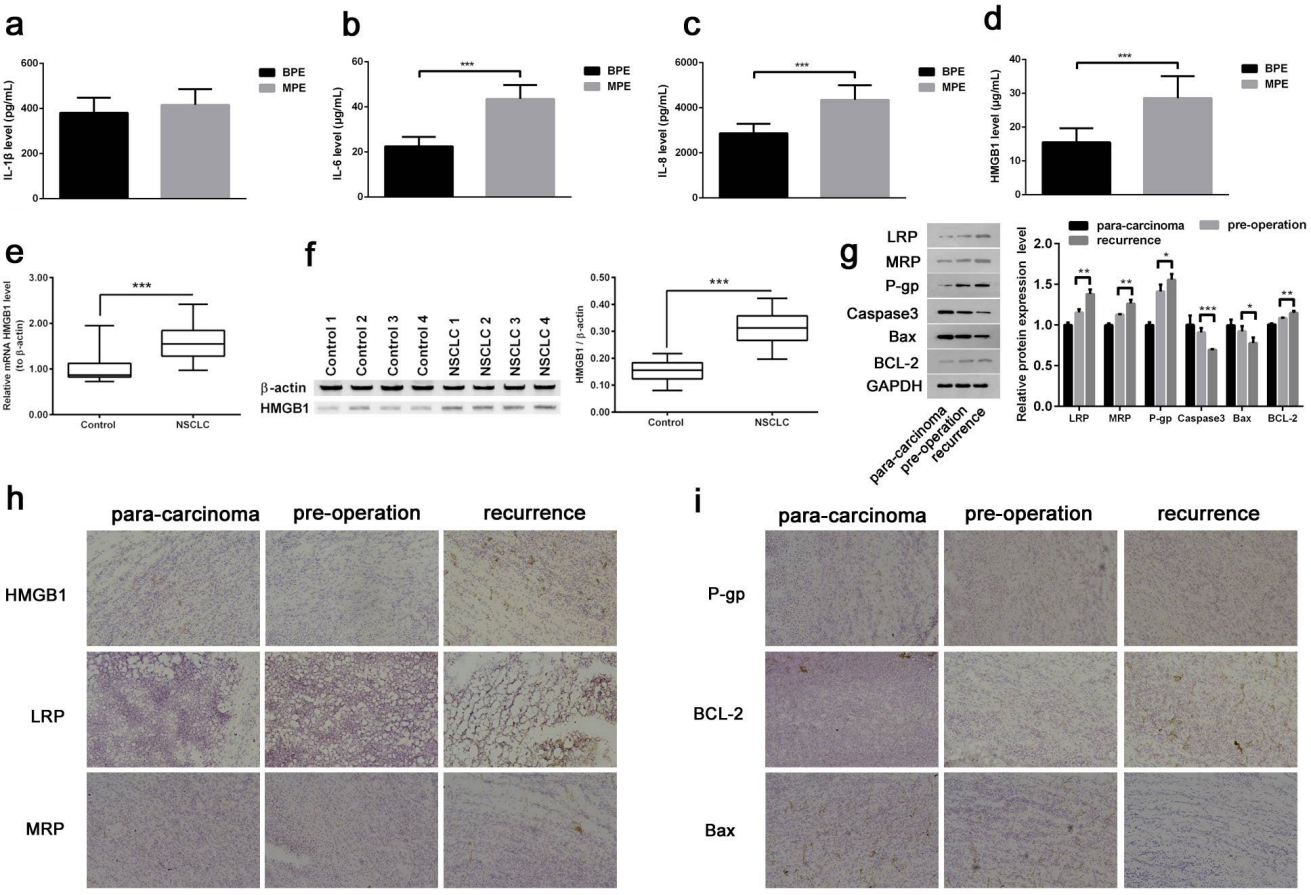
The HMGB1 (high mobility group box B-1) expression in pleural effusion and non-small cell lung cancer (NSCLC) samples. (**a**-**d**) The expression of proinflammatory factors, IL-1β, IL-6, IL-8, and HMGB1 in MPE vs. BPE by ELISA. (**e**-**f**) The mRNA and protein expression of HMGB1 in NSCLC were detected by qRT-PCR (**e**) and Western blot (**f**). (**g**) Western blot was performed to detect the expression of LRP, MRP, P-gp, Caspase, Bax and Bcl-2. (**h**-**i**) Immunohistochemical assay was performed to detect the expression of HMGB1, LRP, MRP, P-gp, Bax and Bcl-2 in NSCLC samples. Magnification, ×200. * P < 0.05, ** P < 0.01 and *** P < 0.001

To evaluate the potential correlation between HMGB1 and the malignant progression of NSCLC, the HMGB1 expression in pleural effusion samples with NSCLC as well as patients’ clinicopathological features were jointly analyzed. All patients’ clinical information were summarized in Table 1, which indicated that there was no statistically significant variation in age, sex, or smoking status between MPE and BPE samples. There were also no appreciable differences in lung small cell carcinomas regarding age, gender, smoking status, or HMGB1 concentration (Table 2). However, lung adenocarcinoma samples showed a substantially greater HMGB1 concentration than lung squamous cell carcinoma. Moreover, IL-1β showed no significant difference between MPE vs. BPE (Fig. 1a), while there were significantly higher IL-6, IL-8, and HMGB1 levels in MPE group compared with BPE group (Fig. 1b and d).

**Table 1:** The clinicopathological features of all pleural effusion cases

Characteristic	MPE (n = 46)	BPE (n = 31)	P value
Age, years	61.45 ± 9.52	63.70 ± 10.55	0.430
Sex			0.336
Male	27	14	
Female	21	17	
Smoking			0.085
Yes	27	12	
No	19	19	
Types			
Adenocarcinoma	38	-	
Squamous cell	8	-	
Carcinoma			

MPE: malignant pleural effusion; BPE: non-malignant pleural effusion

**Table 2:** The clinicopathological features of all NSCLC patients

Characteristic	Numbers	HMGB1 (µg/L)	P value
Age, years			0.306
≤ 55	17	29.30 ± 12.01	
> 55	29	24.99 ± 9.82	
Sex			0.471
Male	27	23.69 ± 10.16	
Female	21	26.92 ± 12.13	
Smoking			0.103
Yes	27	27.96 ± 12.35	
No	19	24.40 ± 10.46	
Types			0.016
Adenocarcinoma	38	28.59 ± 10.71	
Squamous cell carcinoma	8	19.54 ± 12.08	

Values are mean ± SD.

### Significantly higher HMGB1 expression in NSCLC was correlated with tumor recurrence

Next, the HMGB1 mRNA and protein expression was also analyzed in NSCLC. Our results showed that the mRNA and protein expression of HMGB1 were both significantly upregulated in NSCLC samples compared to adjacent samples (Fig. 1e and f). To study the correlation between HMGB1 and different stages of NSCLC, the expression levels of HMGB1 protein, drug-resistant related proteins and apoptosis-related proteins were examined in adjacent samples, pre-operation NSCLC and recurrence NSCLC samples, respectively. Compared with pre-operation NSCLC samples, the drug-resistant proteins LRP, P-gp, and MRP were significantly upregulated in recurrence NSCLC samples (Fig. 1g). The apoptosis marker proteins, Caspase 3 and Bax, showed significantly decreased levels, while proliferation marker protein Bcl-2 showed higher level in recurrence NSCLC samples than pre-operation NSCLC samples (Fig. 1g). Moreover, IHC results exhibited similar expression tendency of these proteins. Significantly higher HMGB1, LRP, MRP, P-gp, and Bcl-2 expression levels while lower Bax expression were observed in recurrence NSCLC samples compared to pre-operation NSCLC samples (Fig. 1h and i). Thus, the elevated HMGB1 expression was probably correlated with the recurrence of NSCLC.

### HMGB1 promoted cisplatin resistance of lung cancer cells

**Fig. 2:**
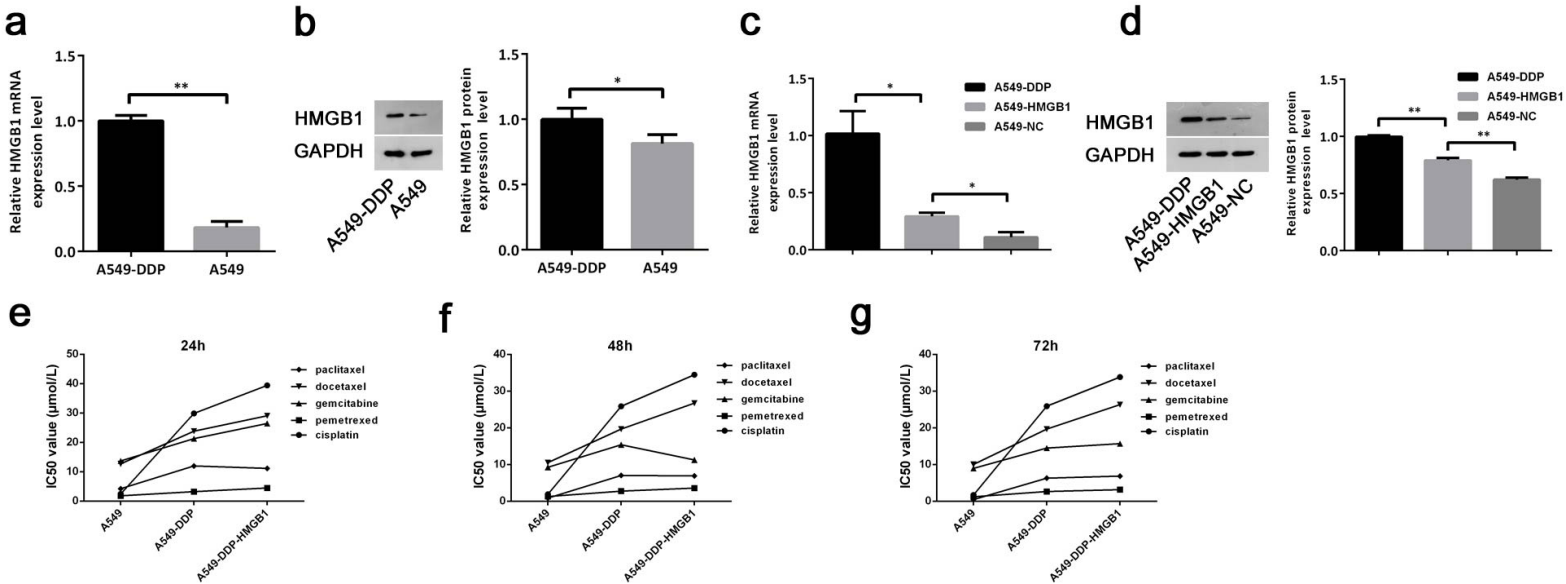
HMGB1 promoted cisplatin resistance of A549 cells. (**a**-**b**) The mRNA and protein expression of HMGB1 in A549 and A549-DDP were detected by qRT-PCR (**a**) and Western blot (**b**). (**c**-**d**) A549 cells were transfected with NC or HMGB1. Next, western blot was used to determine HMGB1 expression in A549-DDP cells and in transfected A549 cells. qRT-PCR (c) and Western blot (d) were used to determine the interference efficiency of HMGB1. (e-g) IC50 values of paclitaxel, docetaxel, gemcitabine, pemetrexed, and cisplatin, in three cell lines after 24 h, 48 and 72 h treatment, separately. * P < 0.05, ** P < 0.01 and *** P < 0.001

Furthermore, the potential association of HMGB1 and drug resistance in NSCLC was analyzed in A549 cells and A549-DDP cells. We found that HMGB1 mRNA and protein expression level in cisplatin-resistant NSCLC A549-DDP cells were significantly higher than that in A549 cells (Fig. 2a and b). Subsequently, a HMGB1 overexpression vector was constructed in A549 cells, and the results suggested that the expression of HMGB1 in A549-DDP was significantly higher than that in HMGB1-overexpressed A549 cells (Fig. 2c and d).

Then, 5 types of common chemotherapy drugs, paclitaxel, docetaxel, gemcitabine, pemetrexed, and cisplatin, were used to administrate the cells, in order to evaluate the drug resistance of various cells. After 24 h of drug treatment, there were significantly differential IC50 values of various drugs in A549, A549-DDP, and HMGB1-overexpressed A549-DDP cells, meanwhile most of the IC50 value tendency was consistent with that in cells after 48 and 72 h’s treatment (Fig. 2e g, Table S2-S3, Fig S1). In all three treatment groups (24 h, 48 h, 72 h treatment), HMGB1-overexpressed A549-DDP cells showed the highest cisplatin resistance among three cell lines. In addition, except for paclitaxel, the IC50 values of other drugs in HMGB1-overexpressed A549-DDP cells and A549-DDP were significantly different (Fig. 2e g). Therefore, MTT was also used to determine the correlation between cisplatin and paclitaxel resistance index under 48 h stimulation in subsequent analysis. Collectively, our data indicated that higher HMGB1 expression could promote the drug resistance of A549 cells, especially cisplatin resistance.

### Elevated HMGB1 promoted the malignant transformation of NSCLC cells

**Fig. 3:**
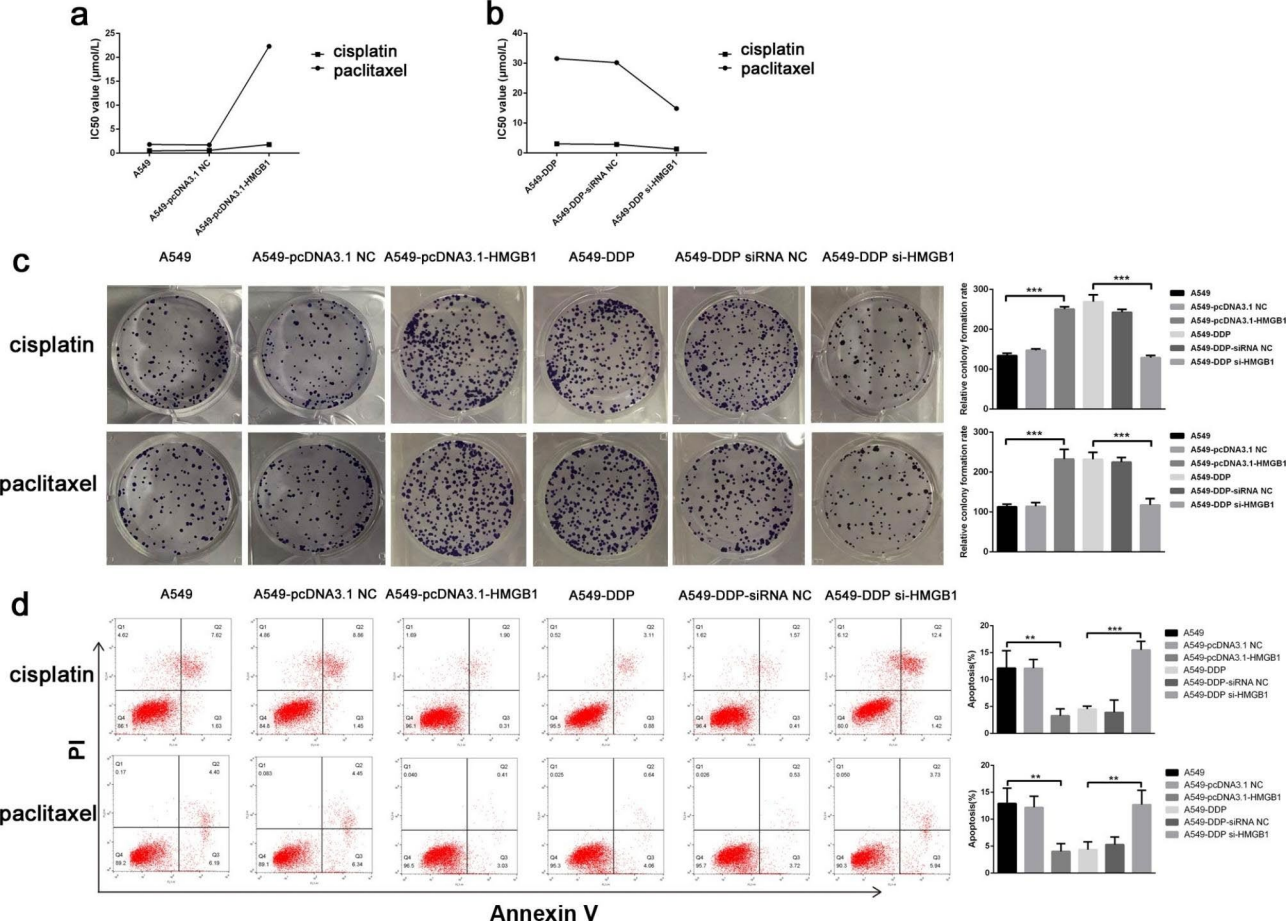
HMGB1 promoted the malignant transformation of A549 cells and A549-DDP cells. (**a**-**b**) IC50 values of cisplatin and paclitaxel treatment on A549 cells and A549-DDP cells. (**c**) Clony formation assay was used to determine the cells’ proliferative ability. (**d**) Flow cytometry was performed to measure the apoptosis rates of cells. * P < 0.05, ** P < 0.01 and *** P < 0.001

To confirm whether HMGB1 contributed to the carcinogenesis and malignant transformation of NSCLC cells, further in vitro study was then conducted. The IC50 values of both cisplatin and paclitaxel treatment groups proliferated greatly when HMGB1 was overexpressed, although the cisplatin’s impact was more pronounced (Fig. 3a), implying the enhancing drug resistance. Whereas, the IC50 values of cisplatin and paclitaxel significantly decreased in A549-DDP-si-HMGB1 cells (Fig. 3b). Above results suggested that HMGB1 was more likely to affect the sensibility of cisplatin. Moreover, clone formation assay and flow cytometry experiment were performed to explore HMGB1 expression’s effects on NSCLC cell proliferation. We found that HMGB1 overexpression enhanced the proliferative capacity of A549 cells under cisplatin and paclitaxel treatment, whilst A549-DDP cell proliferation was dramatically reduced in the cisplatin group when HMGB1 was silenced (Fig. 3c). The A549 and A549-DDP cell apoptosis results were consistent with cell proliferation (Fig. 3d).

**Fig. 4:**
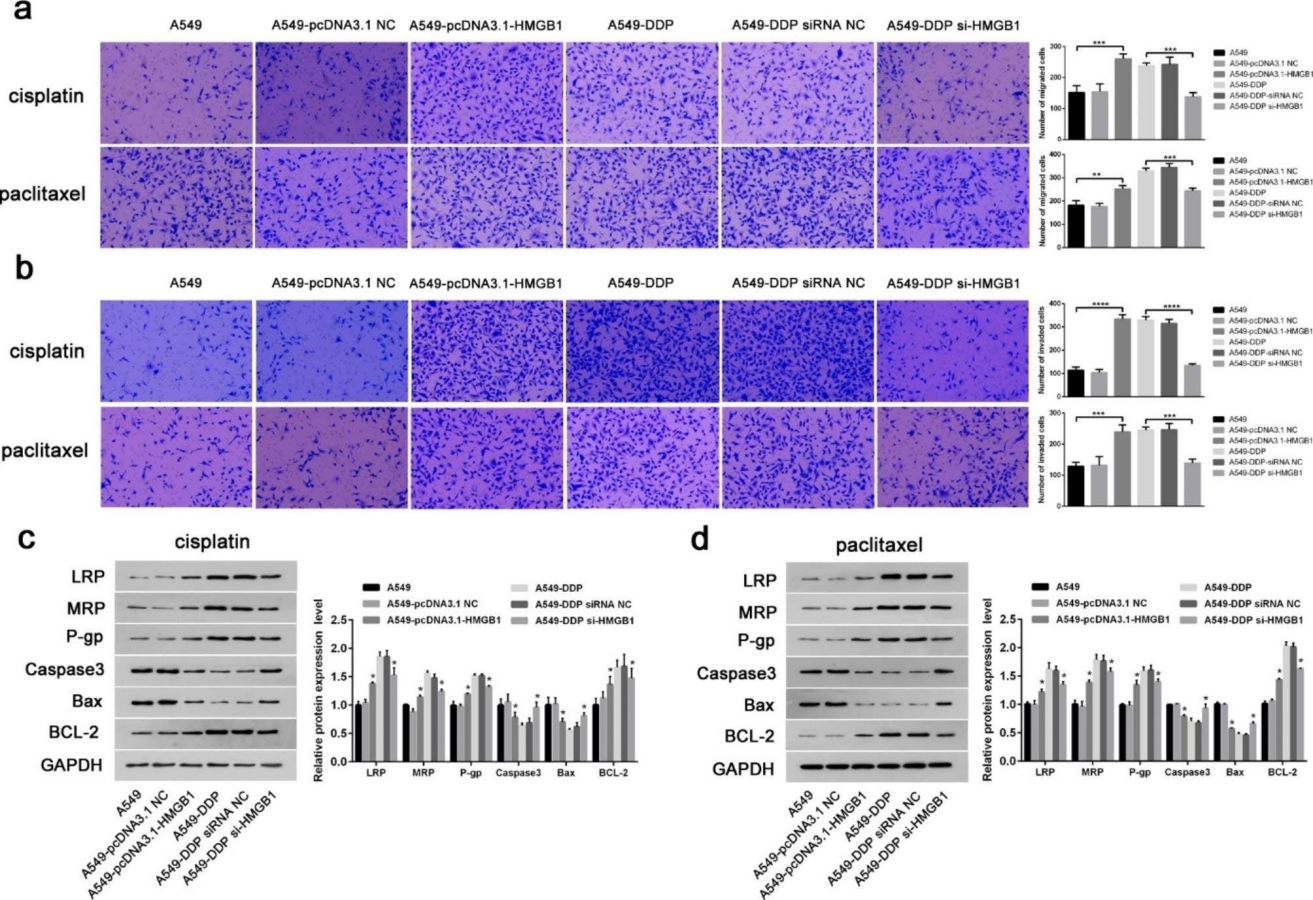
HMGB1 promoted the migration, invasion and drug resistance of A549 cells and A549-DDP cells. (**a**) Transwell assay was conducted to determine the effects of HMGB1 on the migration ability of A549 cells and A549-DDP cells. Magnification, ×200. (**b**) Transwell assay was conducted to detect the effect of HMGB1 on the invasive ability of A549 cells and A549-DDP cells. Magnification, ×200. (**c**-**d**) Western blot was performed to detect the expression levels of LRP, MRP, P-gp, Caspase, Bax and Bcl-2 in A549 cells and A549-DDP cells under cisplatin and paclitaxel treatment, respectively. *P < 0.05, **P < 0.01 and ***P < 0.001

Additionally, Transwell analysis of cell migration and invasion demonstrated that overexpressed HMGB1 promoted the migration and invasion ability of A549 cells under cisplatin and paclitaxel treatment (Fig. 4a and b). The reverse results were seen when HMGB1 was suppressed, and the cell migration and invasion of A549-DDP cells were significantly inhibited. Finally, we confirmed the drug resistance and apoptosis level of cells treated with cisplatin or paclitaxel by Western blot assay. Overexpression of HMGB1 promoted the expression of drug-resistant and proliferative marker proteins, but silenced HMGB1 improved the sensitivity of drug-resistant cells and promoted cell apoptosis (Fig. 4c and d). Thus, HMGB1 overexpression promoted the malignant transformation of NSCLC cells.

### HMGB1 overexpression promoted the malignant progression and cisplatin resistance of NSCLC xenograft model

**Fig. 5:**
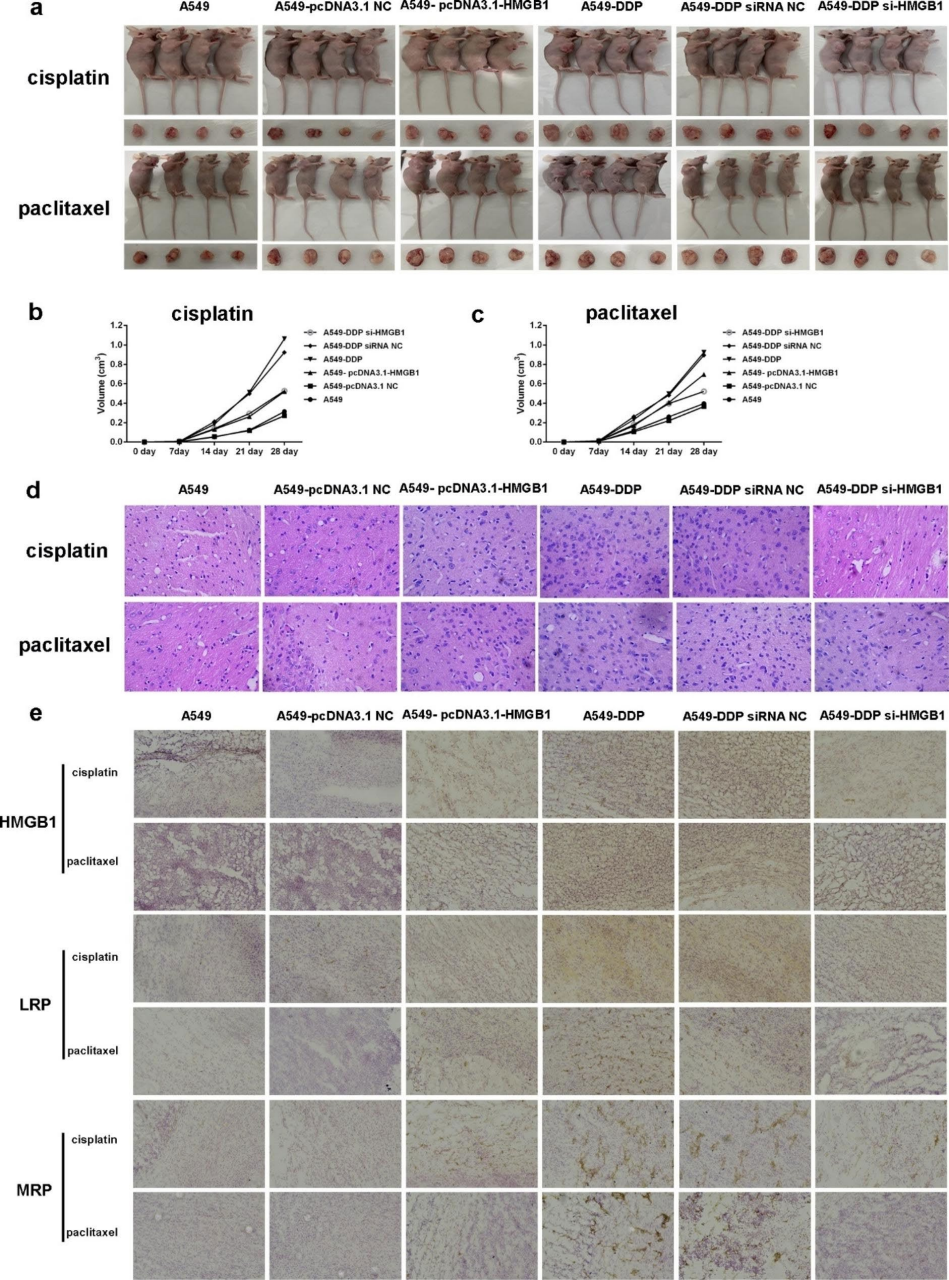
HMGB1 overexpression promoted the malignant progression in vivo. (**a**) Tumorigenesis experiment in nude mice with cisplatin and paclitaxel treatment. (**b**-**c**) Tumor volume curves of mice with cisplatin and paclitaxel treatment, respectively. (**d**) Representative HE staining results. Magnification, ×200. (**e**) Representative IHC results of the expression levels of HMBG1, LRP and MRP after cisplatin and paclitaxel treatment. Scale bar, 100 μm

In order to better understand the function of HMGB1 in the drug resistance of NSCLC, we established the mouse xenograft model with subcutaneously injecting A549, A549-pcDNA3.1-NC, A549-pcDNA3.1-HMGB1, A549-DDP, A549-DDP-siRNA-NC, and A549-DDP-si-HMGB1 cells. After 28 days, overexpression of HMGB1 remarkably promoted tumor growth, while the tumor volume of A549-DDP-si-HMGB1 mice was smaller than that of A549-DDP group (Fig. 5a and c, Table S4). HE staining results showed that with cisplatin and paclitaxel treatment, the tumor nuclei in A549-pcDNA3.1-HMGB1 mice were large and stained deeply, and the cell density was high. In A549-DDP-si-HMGB1 group, the tumor cell apoptosis level increased, the number of cell shrinkage was the largest, and the necrosis area was larger (Fig. 5d).

**Fig. 6:**
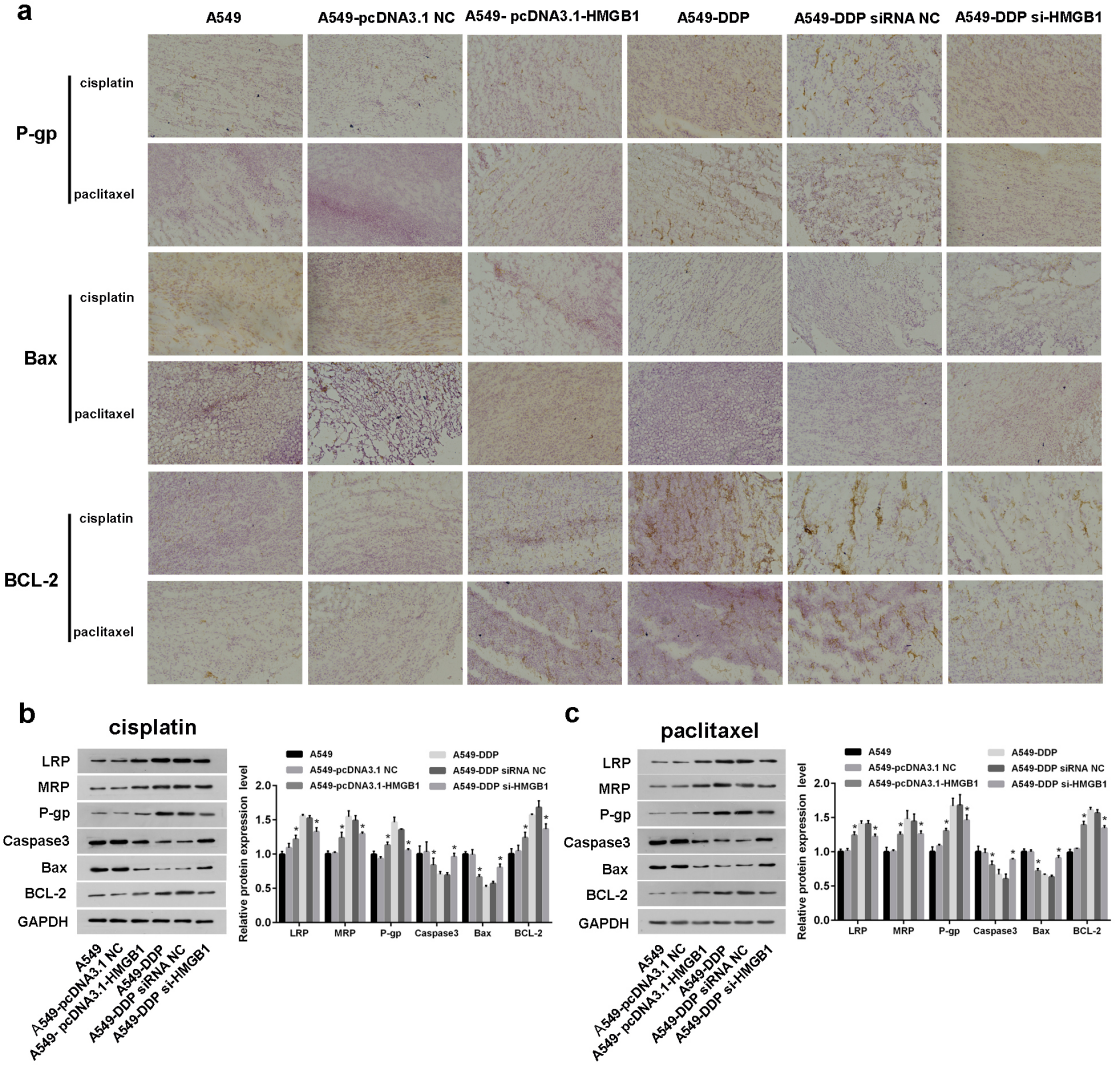
HMGB1 overexpression promoted the cisplatin resistance in vivo. (**a**) Representative IHC results of the expression levels of P-gp, Bax and BCL-2 after cisplatin and paclitaxel treatment. Scale bar, 100 μm. (**b**-**c**) Western blot was performed to detect the expression of LRP, MRP, P-gp, Caspase, Bax and Bcl-2. * P < 0.05, ** P < 0.01 and *** P < 0.001

**Fig. 7:**
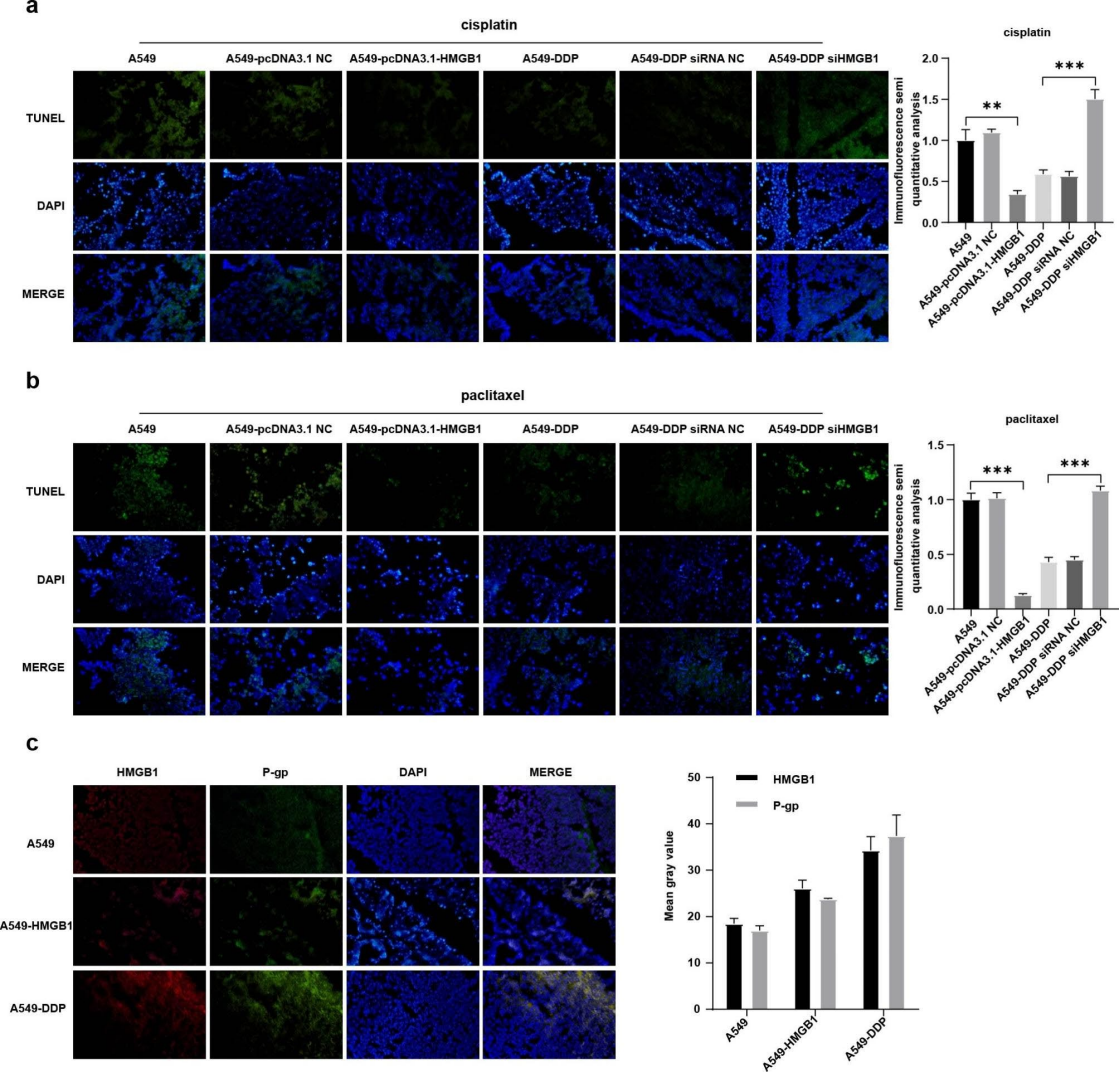
HMGB1 overexpression promoted tumor proliferation and co-localized with P-gp protein in vivo. (**a**-**b**) After cisplatin and paclitaxel therapy, separately, the tumor cell apoptosis was determined using the TUNEL assay. Blue: nucleus; green: cell apoptosis level. Magnification, ×200. (**c**) Immunofluorescence assay was used to detect co-localization of HMGB1 and P-gp proteins. Blue: nucleus; red: HMGB1 expression; green: P-gp expression. Base magnification: ×200. *P < 0.05, **P < 0.01 and ***P < 0.001

Then, the expression levels of HMGB1, drug-resistant proteins, apoptosis marker proteins, and proliferation marker protein in tumor tissues were also analyzed using IHC. Compared with A549 and A549-pcDNA3.1-NC groups, significantly higher HMGB1 protein expression level was observed in A549-PCDNA3.1-HMGB1 group (Figs. 5e and 6a). In HMGB1 overexpressed group, the expression levels of Bcl-2, LRP, MRP, and P-gp were increased, while Bax were decreased compared to control group (Figs. 5e and 6a). When HMGB1 was interfered, the expression of these crucial proteins exhibited an opposite trend (Figs. 5e and 6a). Meanwhile, Western blot and TUNEL assay were also employed to confirm the malignant progression promoting role of HMGB1 overexpression on NSCLC, which was content with our above results (Figs. 6b and c and 7a and b). Our results implied that HMGB1 overexpression promoted the drug resistance but suppressed the tumor cell apoptosis in NSCLC xenograft model.

After the immunofluorescence assay, we found that HMGB1 mainly co-localized with nucleus in A549 mouse model, while in A549-DDP mouse model, HMGB1 displayed similar cytoplasm co-localization with drug resistant transmembrane protein P-gp (Fig. 7c). Our results suggested that HMGB1 overexpression was mainly localized in cytoplasm, with a similar co-localization with P-gp, but low HMGB1 expression predominantly co-localized with nucleus. Collectively, our findings implied that HMGB1 overexpression probably exerted a drug resistant role and malignant development promoting role in NSCLC via the altered cell localization in tumor cells.

## Discussion

As a leading cause of cancer related morbidity and mortality, lung cancer has been endangering human life and health for years. Moreover, lung cancer is often accompanied by metastasis due to lacking of rapid effective diagnosis method as well as coupling with the instability of NSCLC [23]. Thus, it is of great significance to systematically investigate the mechanisms underlying the malignant progression of NSCLC, thereby improve the treatment strategies of patients. In this work, we have predominantly revealed the malignant progression promoting role of HMGB1 overexpression, meanwhile overexpressed HMGB1 also contributed to the cisplatin resistance of NSCLC in vitro and in vivo.

MPE frequently coexists with the advanced NSCLC, therefore the correlation between of HMGB1 expression and MPE was explored herein. Regarding age, gender, and smoking status, we found that there was no statistically significant difference between MPE vs. BPE in this research. The pleural effusion has been indicated to associate with inflammation of the pleura caused by the invasion of tumor cells [24, 25]. The activation of inflammatory cells mainly results from the tissue necrosis caused by tumor cells, and necrotic cells release inflammatory factors to activate neutrophils. Mononuclear macrophages and other inflammatory mediators could activate inflammatory cells [26–28]. Our data also showed consistent results that there were higher levels of proinflammatory factors in MPE group compared with BPE samples.

Chemotherapeutic drug resistance is the biggest obstacle for the recovery of NSCLC patients [29]. Multi-drug resistance (MDR) in lung cancer refers to the phenomenon that the cross-resistance to a variety of unused chemotherapy drugs was caused by a single chemotherapy drug, the related proteins of which mainly included MDR1, MRPs, P-gp, and LRP [30–32]. The mechanism behind multidrug resistance in lung cancer is quite complex, which remains largely unknown. In this study, we found a favorable correlation between HMGB1 expression and drug resistance, malignant progression of NSCLC. In addition, the up-regulation of HMGB1 in the mouse model tissues also promoted the expression of drug-resistant related proteins and proliferative marker proteins.

Elevated HMGB1 in tumor cells has been correlated with tumor formation, cell proliferation and metastasis, and chemotherapy response [33, 34]. Previous studies have demonstrated that HMGB1 interfered with cyclin expression through a variety of downstream pathways to maintain cell growth, or through binding to surface RAGE receptors to enhance the metalloproteinases activity to promote tumor invasion and metastasis [35]. In our study, cisplatin-resistant cell line A549/DDP showed a much greater level of HMGB1 than cisplatin-sensitive cell line A549. This was consistent with the study of Zheng et al., which detected HMGB1 expression changes in the A549 cell line through in vitro culture [36]. According to previous reports, in HMGB1-knockouted A549 cells, cell proliferation ability, biological activity and activity function were significantly reduced [37]. The expression level of HMGB1 showed a positive linear relationship with chemotherapy resistance.

Earlier study has reported that the serum HMGB1 expression level also could serve as a key indicator, and HMGB1 was associated with a good prognosis during late prognostic chemotherapy treatment [38]. In this study, we constructed a tumor-forming model in nude mice, and revealed that after HMGB1 interference, cell morphology changed, nucleo-plasma specific gravity decreased, wrinkling status appeared, drug-resistant protein expression decreased, and cells showed obvious apoptosis and necrosis. Suppressing HMGB1 expression down-regulated the molecular level and activity of Bcl-2, blocked the binding of Bcl-2 to autophagy gene Beclin1, and inhibited autophagy, thus promoted the apoptosis of tumor cells [39]. The process outlined above was congruent with the experimental findings of our investigation.

P-gp is able to be activated by binding with ATP through nucleotide binding sites, which can transfer cytotoxic drugs to the extracellular, thereby weakening or even completely losing the cytotoxic effect of drugs, and enhancing drug resistance. Related research has demonstrated that individuals receiving anticancer chemotherapy medications showed considerably greater P-gp protein expression level than untreated patients. In addition, the survival rate of highly expressed P-gp patients was negatively correlated the P-gp protein expression during the treatment process [40, 41]. HMGB1 is primarily expressed in the nucleus in the early stage, and it would play the role of extracellular inflammatory mediator mainly through two pathways, including active release of mononuclear macrophages and passive release of cell rupture [30]. After tumor cells are stimulated, classical protein kinases and calmodulin-dependent protein kinases are activated to stimulate HMGB1 metastasis [42]. In addition, the methylation modification of HMGB1 made it be free from the nucleus, then HMGB1 was transferred to the cytoplasm and accumulated in the cytoplasm, meanwhile the HMGB1 cannot return to the nucleus [43]. On this point, our results of immunofluorescence assay showed consistent potential, we found that HMGB1 overexpression was mainly localized in cytoplasm, with a similar co-localization with P-gp, but low HMGB1 expression predominantly co-localized with nucleus. We suspected that along with the expression alteration of HMGB1, HMGB1 might change from structure maintaining to promoting cell proliferation or diffusion in the progression of NSCLC. More details associating with HMGB1 and P-gp in NSCLC deserved further exploration in the near future.

Herein, we have extensively analyzed the role of HMGB1 in NSCLC cell migration, invasion, proliferation, and apoptosis, as well as drug resistance, whereas there were still some limitations in our study. Predominantly, this work has focused on the functional role of HMGB1 in NSCLC, while its related functional axis or signaling pathways need to be further investigated in our future work.

## Conclusions

To summarize, our present work has mainly explored the crucial role of HMGB1 in NSCLC cell migration, invasion, proliferation, and apoptosis, as well as drug resistance, employing in vitro and in vivo methods. HMGB1 overexpression significantly promoted the malignant progression of NSCLC, meanwhile it also contributed to the cisplatin resistance of NSCLC. Overexpressed HMGB1 showed a similar co-localization with P-gp in cytoplasm, which probably explained its drug resistant role in NSCLC. Our novel evidences regarding HMGB1 in NSCLC provide more reference information not only for understanding cisplatin resistance but also for promising therapeutic targets of NSCLC patients.

## Electronic supplementary material

Below is the link to the electronic supplementary material.

Supplementary Material 1

Supplementary Material 2

Supplementary Material 3

Supplementary Material 4

Supplementary Material 5

Supplementary Material 6

## Data Availability

Data for this study is available from the corresponding author upon reasonable request.

## Abbreviations

HE: Hematoxylin-eosin
IHC: Immunohistochemistry
NSCLCs: Non-small cell lung cancers
PVDF: Polyvinylidene difluoride
qRT-PCR: Quantitative Real-Time Polymerase Chain Reaction
RT: Room temperature
