# Supplementary figures and images for: Elevated HMGB1 promotes the malignant progression and contributes to cisplatin resistance of non-small cell lung cancer
Source: Hereditas. 2023 Jul 31;160:33. doi: 10.1186/s41065-023-00294-9 (PMC10388484; doi:10.1186/s41065-023-00294-9)

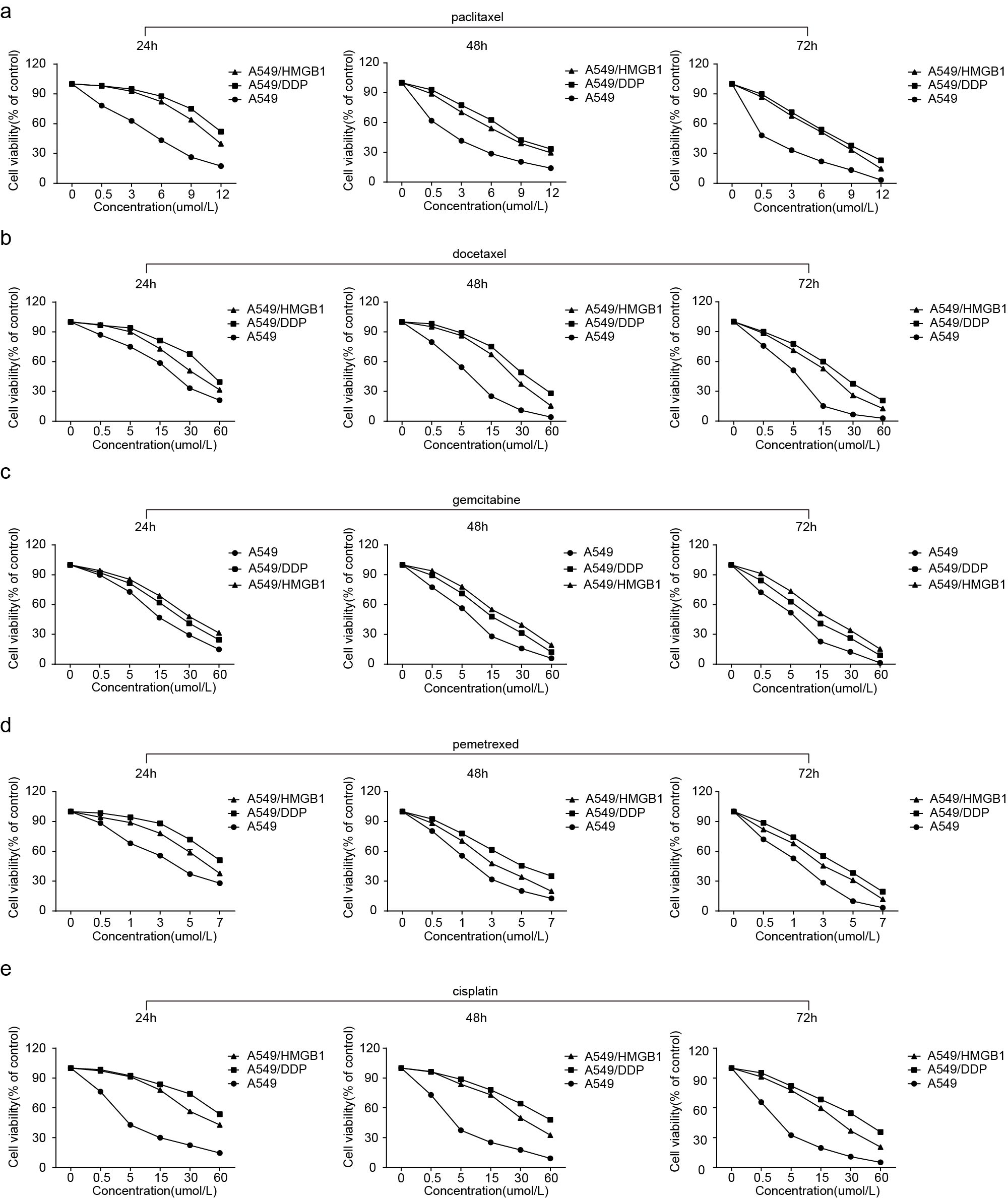

Supplement: Supplementary file 1 — Supplementary Material 1 [file 41065_2023_294_MOESM1_ESM.jpg]

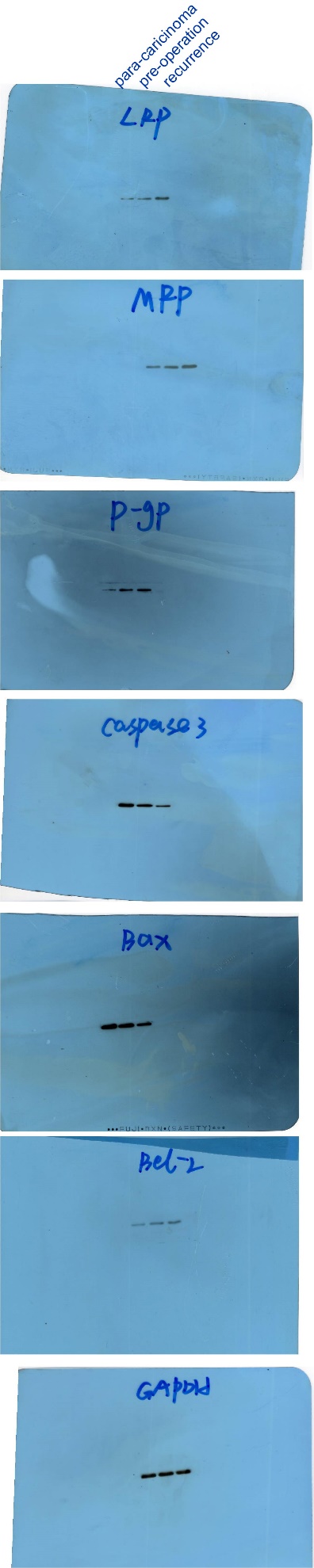

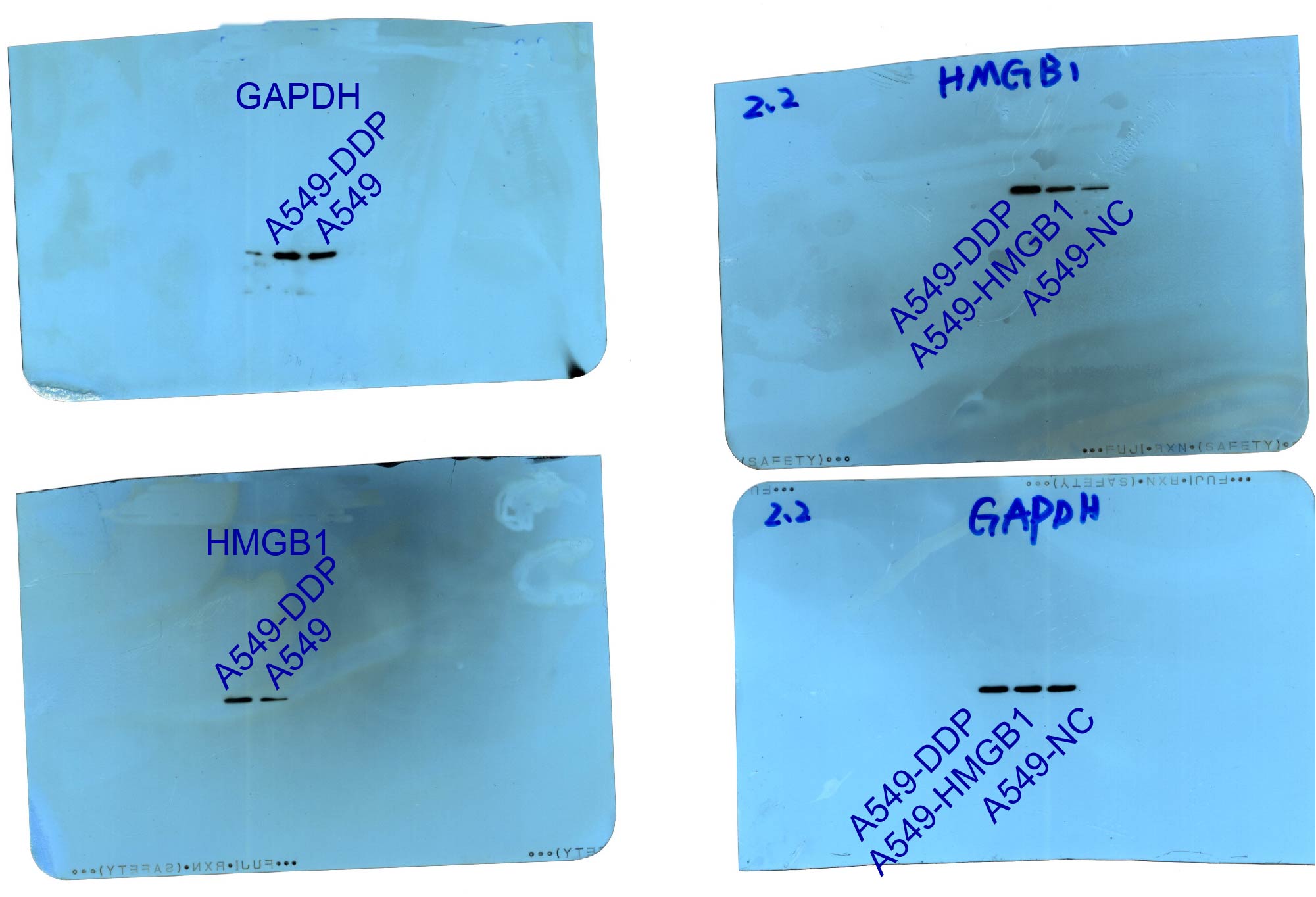

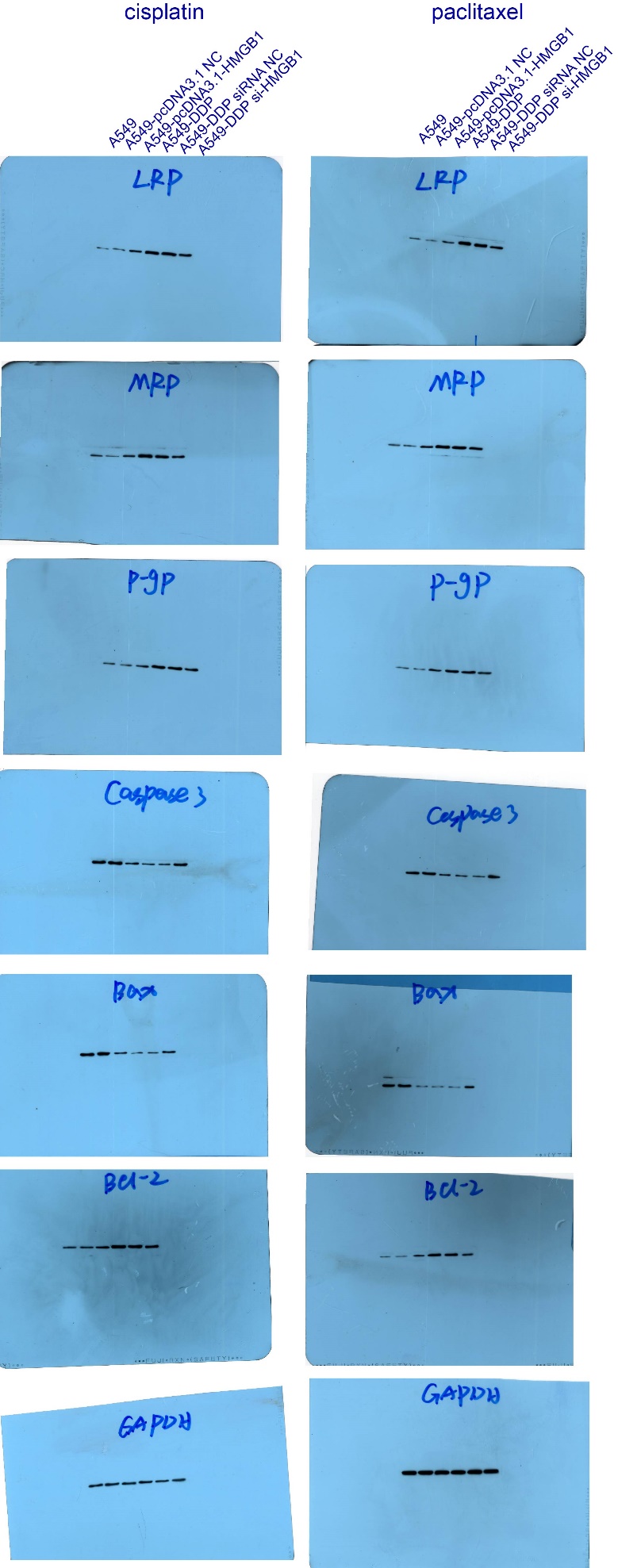

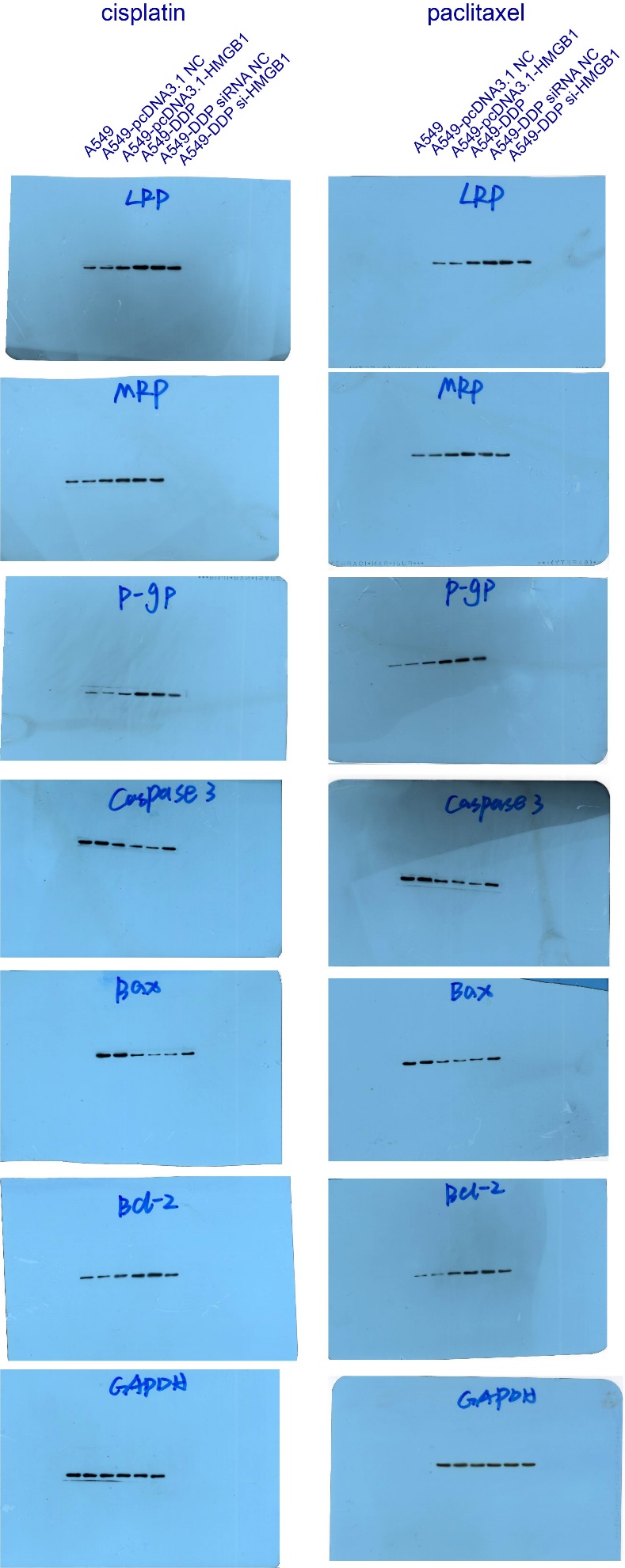

Supplement: Supplementary file 6 — Supplementary Material 6 [file 41065_2023_294_MOESM6_ESM.docx]
