# Supplementary material for: Elevated HMGB1 promotes the malignant progression and contributes to cisplatin resistance of non-small cell lung cancer
Source: Hereditas. 2023 Jul 31;160:33. doi: 10.1186/s41065-023-00294-9 (PMC10388484; doi:10.1186/s41065-023-00294-9)
Supplement: Supplementary file 2 — Supplementary Material 2 [file 41065_2023_294_MOESM2_ESM.docx]

Table S1 Primer sequences.

| Gene^*^ | Product Size (bp) | Primer Pair | Primer Sequence (5’-3’) |
| --- | --- | --- | --- |
| HMGB1 | 210 | Forward | ATGCGCAAAGGAGATCCTA |
|  |  | Reverse | ATTCATCATCATCTTCT |
| GAPDH | 200 | Forward | ACAGTCAGCCGCATCTTCTT |
|  |  | Reverse | GACAAGCTTCCCGTTCTCAG |
