# Supplementary material for: Elevated HMGB1 promotes the malignant progression and contributes to cisplatin resistance of non-small cell lung cancer
Source: Hereditas. 2023 Jul 31;160:33. doi: 10.1186/s41065-023-00294-9 (PMC10388484; doi:10.1186/s41065-023-00294-9)
Supplement: Supplementary file 3 — Supplementary Material 3 [file 41065_2023_294_MOESM3_ESM.docx]

Table S2. MTT detected the IC_50_ of cells to different drugs at 24h, 48h and 72h.

| Medicine/IC50 | 24 h | | | 48 h | | | 72 h | | |
| --- | --- | --- | --- | --- | --- | --- | --- | --- | --- |
|  | A549 | A549-HMGB1 | A549/DDP | A549 | A549-HMGB1 | A549/DDP | A549 | A549-HMGB1 | A549/DDP |
| cisplatin | 4.132 | 43.82 | 59.77 | 1.88 | 25.86 | 34.46 | 1.697 | 25.9 | 33.81 |
| gemcitabine | 13.68 | 26.43 | 21.26 | 9.262 | 15.37 | 11.24 | 8.957 | 15.69 | 14.47 |
| docetaxel | 17.1 | 32.05 | 40.25 | 7.567 | 19.65 | 26.70 | 6.675 | 19.64 | 26.32 |
| pemetrexed | 3.594 | 5.705 | 7.094 | 1.30 | 2.761 | 3.587 | 1.208 | 2.66 | 3.166 |
| paclitaxel | 4.241 | 11.16 | 11.97 | 0.8537 | 6.919 | 7.033 | 0.4557 | 6.293 | 6.862 |
