# Supplementary material for: Elevated HMGB1 promotes the malignant progression and contributes to cisplatin resistance of non-small cell lung cancer
Source: Hereditas. 2023 Jul 31;160:33. doi: 10.1186/s41065-023-00294-9 (PMC10388484; doi:10.1186/s41065-023-00294-9)
Supplement: Supplementary file 4 — Supplementary Material 4 [file 41065_2023_294_MOESM4_ESM.docx]

Table S3. MTT detected the resistance index of cells to different drugs at 24 h, 48h and 72h.

| Resistance index | 24 h | | 48 h | | 72 h | |
| --- | --- | --- | --- | --- | --- | --- |
|  | A549-HMGB1 | A549/DDP | A549-HMGB1 | A549/DDP | A549-HMGB1 | A549/DDP |
| cisplatin | 10.605 | 14.465 | 13.756 | 18.330 | 15.262 | 19.923 |
| gemcitabine | 1.932 | 1.554 | 1.659 | 1.213 | 1.751 | 1.615 |
| docetaxel | 1.874 | 2.353 | 1.869 | 2.541 | 1.964 | 2.632 |
| pemetrexed | 1.587 | 1.973 | 2.124 | 2.760 | 2.202 | 2.621 |
| paclitaxel | 2.631 | 2.822 | 8.104 | 8.238 | 13.809 | 15.058 |

Drug resistance index = mean IC50 of drug-resistant cell lines/ mean IC50 of sensitive cell lines.
