# Supplementary material for: Elevated HMGB1 promotes the malignant progression and contributes to cisplatin resistance of non-small cell lung cancer
Source: Hereditas. 2023 Jul 31;160:33. doi: 10.1186/s41065-023-00294-9 (PMC10388484; doi:10.1186/s41065-023-00294-9)
Supplement: Supplementary file 5 — Supplementary Material 5 [file 41065_2023_294_MOESM5_ESM.docx]

Table S4. Statistical comparison of tumor size in mice stimulated by cisplatin and paclitaxel

| Group | cisplatin | | | paclitaxel | | |
| --- | --- | --- | --- | --- | --- | --- |
|  | Wide (cm^3^) | Length (cm^3^) | Volume (cm^3^) | Wide (cm^3^) | Length (cm^3^) | Volume (cm^3^) |
| A549 | 0.836±0.031 | 0.902±0.103 | 0.315±0.036 | 0.864±0.076 | 1.055±0.048 | 0.395±0.081 |
| A549-pcDNA3.1 NC | 0.800±0.050 | 0.841±0.072 | 0.271±0.051 | 0.845±0.113 | 0.991±0.111 | 0.365±0.134 |
| A549- pcDNA3.1-HMGB1 | 0.942±0.041 | 1.165±0.103 | 0.519±0.080 | 1.020±0.048 | 1.343±0.109 | 0.696±0.049 |
| A549-DDP | 1.190±0.095 | 1.498±0.184 | 1.063±0.205 | 1.042±0.109 | 1.700±0.086 | 0.926±0.147 |
| A549-DDP siRNA NC | 1.054±0.080 | 1.654±0.141 | 0.924±0.166 | 1.097±0.080 | 1.489±0.094 | 0.894±0.095 |
| A549-DDP si-HMGB1 | 0.941±0.042 | 1.184±0.090 | 0.528±0.083 | 0.874±0.074 | 1.366±0.054 | 0.522±0.069 |
